# The Contribution of Theoretical Prediction Studies to the Antioxidant Activity Assessment of the Bioactive Secoiridoids Encountered in Olive Tree Products and By-Products

**DOI:** 10.3390/molecules28052267

**Published:** 2023-02-28

**Authors:** Nikolaos Nenadis, Ioanna Pyrka, Maria Z. Tsimidou

**Affiliations:** Laboratory of Food Chemistry and Technology, School of Chemistry, Aristotle University of Thessaloniki, 54124 Thessaloniki, Greece

**Keywords:** antioxidant activity, theoretical predictions, phenolic compounds, olive tree products, olive tree by-products, oleuropein, hydroxytyrosol, tyrosol, secoiridoids, density functional theory

## Abstract

Assessment of the antioxidant activity of different types of natural compounds is a complex research area that encompasses various in vitro tests and in vivo studies. Sophisticated modern analytical tools permit the unambiguous characterization of the compounds present in a matrix. The contemporary researcher, knowing the chemical structure of the compounds present, can carry out quantum chemical calculations that provide important physicochemical information assisting the prediction of antioxidant potential and the mechanism behind the activity of target compounds before further experimentation. The efficiency of calculations is steadily improved due to the rapid evolution of both hardware and software. It is possible, consequently, to study compounds of medium or even larger size, incorporating also models that simulate the liquid phase (solution). This review contributes to the establishment of theoretical calculations as an inherent part of the antioxidant activity assessment process, having as a case study the complex mixtures of olive bioactive secoiridoids (oleuropein, ligstroside, and related compounds). The literature indicates great variability in theoretical approaches and models used so far for only a limited number of this group of phenolic compounds. Proposals are made for standardization of methodology (reference compounds, DFT functional, basis set size, and solvation model) to facilitate comparisons and communication of findings.

## 1. Introduction

The interest in natural compounds that exhibit antioxidant activity in food matrices [1] is extended also to the benefits such compounds can exert on humans or animal species after consumption [2,3]. For example, the high oxidative stability of virgin olive oil (VOO) at first was attributed to its rich in monounsaturated fatty acids character and then to the critical role of certain groups of intrinsic antioxidants such as the phenolic compounds present in the polar fraction of the oil. For more than two decades, the interest has been focused on the in vivo properties of the secoiridoids oleuropein, ligstroside, and related derivatives, which can be found not only in the oil but also in other edible products of the olive tree or can be isolated from by-products of the olive industry [4,5]. 

Antioxidants, in general, exhibit their activity even when present in minute concentrations. The phenolic-type antioxidants are considered primary antioxidants because they react directly with reactive oxygen and nitrogen species and have gained the interest of food, medical, pharmaceutical, and cosmeceutical researchers all over the world [6]. The major mechanisms of their action (a–e) have been repeatedly reviewed [7] and are described by reactions (R1) to (R5):

(a) Hydrogen atom transfer (HAT) /Proton coupled electron transfer (PCET) to free radicals:
  AH + ROO^●^ → ROOH + A^●^ (R1)
(b) Single electron transfer followed by proton transfer (SET-PT) or not (SET):
  AH + ROO^●^ → [ROO^−^---AH^●+^ ] → ROOH + A^●^ (R2)
(c) Sequential proton loss followed by electron transfer (SPLET):
  AH + ROO^●^ → A^−^ + ROO^●^ + H^+^ → ROO^−^ + A^●^ + H^+^ → ROOH + A^●^ (R3)
(d) Sequential proton loss−hydrogen atom transfer (SPLHAT):
  AH → A^−^ + H^+^ → A^−^ + ROO^●^ →ROOH + A^●−^ (R4)
(e) Radical adduct formation (RAF):
  AH + ROO^●^ → [AH-OOR]^●^ (R5)

A primary phenolic antioxidant (AH) may react concomitantly with different mechanisms. SET-PT and SPLET mechanisms prevail in polar media, which can facilitate charge separation. In all cases, the derived radical from the antioxidant is expected to be stabilized through electron delocalization, thus being less likely to promote oxidation.

The number of natural phenolic antioxidants is uncountable because of natural variability among and within plant species. Their character is multifunctional, i.e., growth regulators, chemical messengers, or plant tissue defense paraphernalia against mechanical damage, pathogen infection, and UV radiation [8]. Moreover, as secondary plant metabolites, they can be found in free or, mainly, in bound forms. Phenol esters, ethers, glycosides, or bound forms with other biomolecules occur naturally. Structural characteristics influence not only the activity of the compounds in food matrices [9] but also their bioactivity [8]. Moreover, organic synthesis provides a variety of derivatives that can be used in various applications. 

Modern high throughput techniques (e.g., GC or LC coupled to various MS systems) can provide a comprehensive characterization of the compounds present in the analytical sample [10]. Combinations of separation techniques with online antioxidant activity tests served the idea of revealing as much information as possible about the potential of a certain compound, even if its nature was not fully characterized [11]. Nevertheless, such approaches have not found wider acceptance, mainly due to apparent procedural restrictions. All identified compounds cannot be isolated, synthesized, or tested experimentally one by one. Theoretical examination of the antioxidant activity of the individual or selected compounds identified in a matrix can be a green mid-tool for their prioritization regarding antioxidant behavior before seeking experimental in vitro or in vivo data [12]. Such an approach should consider reactions with different free radicals in both gas and phases simulating liquids or lipid media, as nicely presented by Galano and collaborators for sinapinic acid [13]. In vitro studies using synthetic radicals also present limitations, whereas in vivo studies are expensive and time-consuming and are organized only when cumulative in vitro or ex vivo data point out the significance of having an assessment through appropriate intervention and clinical studies, which still, remain scarce [14].

Which theoretical calculations are the most appropriate for such a goal, what they can really offer, which are the most applicable ones for scientists having a basic or advanced quantum chemistry background, how much time is needed to obtain reliable information, what are the actual limitations, and the prospects will be discussed in the next sections and exemplified in detail for the secoiridoid biophenols encountered in olive tree products and by-products. This review aims to contribute to the establishment of theoretical calculations as an inherent part of the antioxidant activity assessment process.

## 2. Theoretical Prediction Strategies

**Figure 1:**
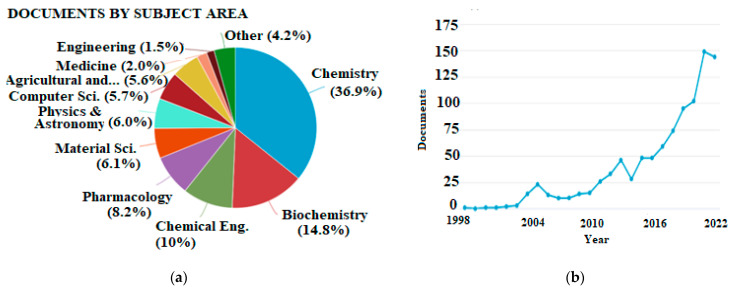
(**a**) Publications categorized by subject area and (**b**) total publications per year appearing in Scopus database using “density functional theory” and “antioxidant” as keywords (accessed 21 November 2022, total documents, 995).

As a result of advances in computer science and software engineering, it is feasible to accomplish theoretical calculations in a reasonable amount of time, making them suitable even for routine studies. The overall number of publications regarding antioxidants that involve theoretical calculations is steadily increasing, but the latter have not found their place in the agriculture and food sector yet. This is indicated by the bibliometrics shown in Figure 1.

In most of the relevant publications, emphasis is given to the study of the radical scavenging process that coincides with the major mechanisms of action of primary antioxidants, such as the phenolic compounds [7,15,16,17,18,19,20,21]. 

Different strategies can be applied to theoretical studies so that the derived data are reported differently, for example, in terms of intrinsic reactivity indices, or using thermochemical or kinetic parameters. In all cases, knowledge of the possible reactions involved and the factors that may influence the reactivity (e.g., environment) is a prerequisite [22].

**Figure 2:**
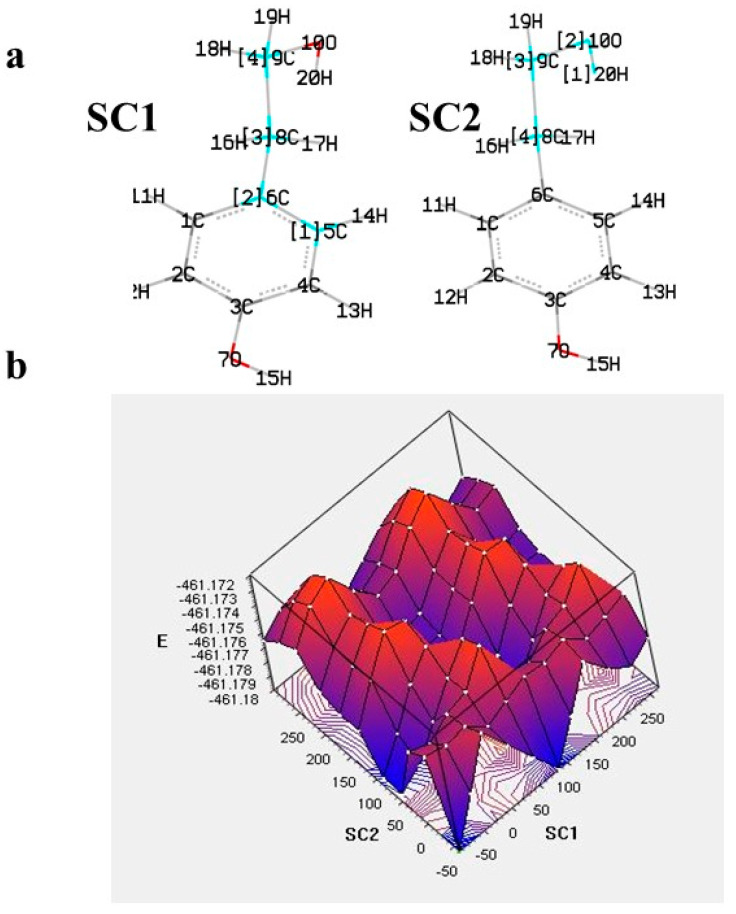
(**a**) The varying dihedrals of tyrosol (SC1: C5-C6-C8-C9, SC2: C8-C9-O10-H20) selected for the potential energy surface (PES) scan at B3LYP/6-31G in gas phase and (**b**) 3D plot of PES [25].

The theoretical approach usually begins with the conformer analysis to locate the most stable compound (global minimum). This requires the examination of all possible structures, often including the determination of enthalpy barriers upon, e.g., rotation of one or more dihedral angles. More elaborate is the potential energy surface (PES) examination, which results in a plot of the energy versus geometry with concomitant rotation of dihedrals. Such an approach can be useful for equilibrium geometries determination and transition states [23,24]. An example of targeted PES examination with a concomitant rotation of two dihedrals is given in Figure 2 for the ligstroside derivative tyrosol. The contour map indicates the place of global minimum, local minima, and destabilization.

Certainly, it is possible to locate more than one conformer, in case the difference in enthalpy from the global minimum is small, suggesting that interconversion can be feasible. Except for the rotation of groups and side chains, tautomerism and shifting from E to Z configuration or bending of dihedral angles are features to be also explored, as highlighted by Spiegel [7]. All of them can affect hydrogen bonding, electron delocalization, polarizability, dipole moment value, as well as steric hindrance phenomena.

Considering that an optimum structure has been obtained, then a first-cut approach resides in the frontier orbital examination. The highest molecular orbital (HOMO) energy is related to electron donation, and the lowest unoccupied molecular orbital (LUMO) to electron acceptance [7,26]. Thus, the energy gap between the two orbitals, electronegativity (χ), chemical potential (μ), global hardness (η), global softness (S), and electrophilicity (ω) can be calculated to have an estimate of molecular reactivity. However, electron correlation (a measure of the influence in an electron movement due to the presence of all other electrons in the system), which is critical for accurate and quantitative evaluation of molecular energies, is not considered and may affect the validity of conclusions [26]. In addition, visualization of HOMO may serve in detecting the structural site of the attack, even though the electrophilic/nucleophilic sites are better revealed via solving the Fukui functionals [27] and more accurately by applying the dual descriptor proposed by Martínez-Araya [28]. 

Another approach is the examination of the so-called intrinsic reactivity of the antioxidants, which is most frequently employed by different research groups [22]. Such an approach requires the calculation of enthalpy of derived species, namely phenoxy radical, cation radical, anion, hydrogen atom, and proton, even of further derivatives depending on whether the structure favors more than one proton or electron donation in a stepwise manner (e.g., double-HAT, double-SPLET, SPLHAT) [21], which in turn are used to calculate the indices given in Table 1.

**Figure 3:**
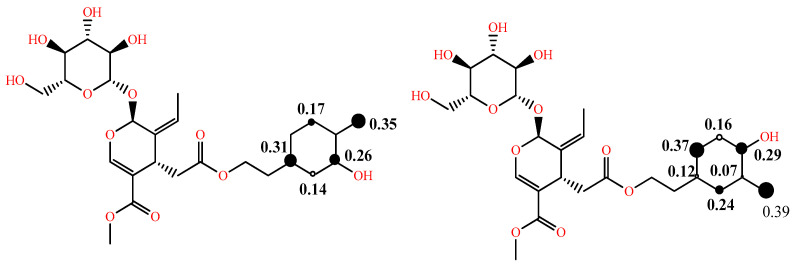
Spin density distribution in phenoxy radicals of oleuropein in the gas phase at B3LYP/6-31G (d,p) level of theory adapted from [30] supplementary; larger size of the black dot corresponds to higher spin density.

In addition to the above, in some studies, to comment on the reactivity and the dominant mechanism, the radical species of interest are also considered, and their BDE and electron affinity (EA) values are computed as well [29]. As the effectiveness of a radical scavenger is not only related to the ease of hydrogen atom and/or electron donation, but also to the stability of the phenoxy radical formed through intramolecular bonding or extension of conjugation, the spin distribution can be obtained and, particularly, the value of the localized spin in the oxygen of the corresponding radical, which can be a useful index of radical stability. The latter is illustrated in Figure 3 for the two phenoxy radicals of oleuropein [30]. Due to the lack of extended conjugation, the electron spin is delocalized only in the aromatic ring. Furthermore, the radical formed from the *p*-OH group is more stable than that from the *m*-OH group, as is evident by the spin value in the oxygen of the corresponding radicals (0.35 vs. 0.39).

When selecting to work from a thermochemical point of view, the calculation of the Gibbs free energy (ΔG) value is the most appropriate index to examine a specific reaction pathway. According to the equation ΔG = Δ*H* − Δ(*TS*), its calculation, except for temperature (T), also includes the entropy (S) of the system that is influenced by the mechanism governing the reaction. The approach is considered to offer more valuable information compared to that obtained from the calculation of indices of intrinsic reactivity, as both the reactivity of the antioxidant and that of a particular free radical species are co-evaluated. The latter is important, considering that the various free radicals to be scavenged differ in reactivity. A drawback, as highlighted by Galano and Alvarez-Idaboy [22], is that when using such a strategy, kinetics is not taken into consideration, and in some cases, opposite trends may be observed due to deviation from pertinence to the Bell–Evans–Polanyi principle, i.e., the usual linear relationship between the activation energy and enthalpy of reaction. Furthermore, it should be stressed that to make feasible the calculation, small-size radicals are usually selected, such as HO^●^, O_2_^●−^, CH_3_OO^•^, and HOO^•^ [31,32].

When a kinetic study is designed for a biological antioxidant, the starting point of consideration is that, according to the definition of Halliwell et al. [33], a compound should react faster with the oxidizing species than the substrate to be considered as a potentially efficient antioxidant. The advantages of kinetic approaches, according to Galano and Alvarez-Idaboy [22], are the following:

- Consideration of tunneling effects for reactions involving small particles;
- Contribution of different mechanisms of reaction, and reaction sites, to the whole activity of the examined compound;
- Inclusion of reactions that do not fulfill the Bell–Evans–Polanyi principle;
- Incorporation of the pH influence on the reactivity of antioxidants bearing ionizable groups such as acids, considering the molar fraction of different forms present when the pH value is different;
- Consideration of single electron transfer reactions situated in the inverted region of the Marcus parabola.

In a kinetic study, except for the overall rate constant of reaction, the contribution of different mechanisms can be estimated via calculation of the corresponding rates to eventually obtain the so-called branching ratios (Γ) according to the following formula:$\;\Gamma =100\frac{ki}{koverall}$, where *ki* represents the rate constant of the mechanism or pathway of interest. A typical example is given below for hydroxytyrosol (Table 2), indicating that the main mechanism involved in the scavenging is the hydrogen atom transfer by ~98% regardless of the polarity of the media. In the lipid media, hydrogen atom transfer from the 1a position was found to be the most preferable, whereas in water, the hydrogen transfer was equally feasible from 1a and 1b due to diffusion limitation [34].

## 3. Computational Methods

**Figure 4:**
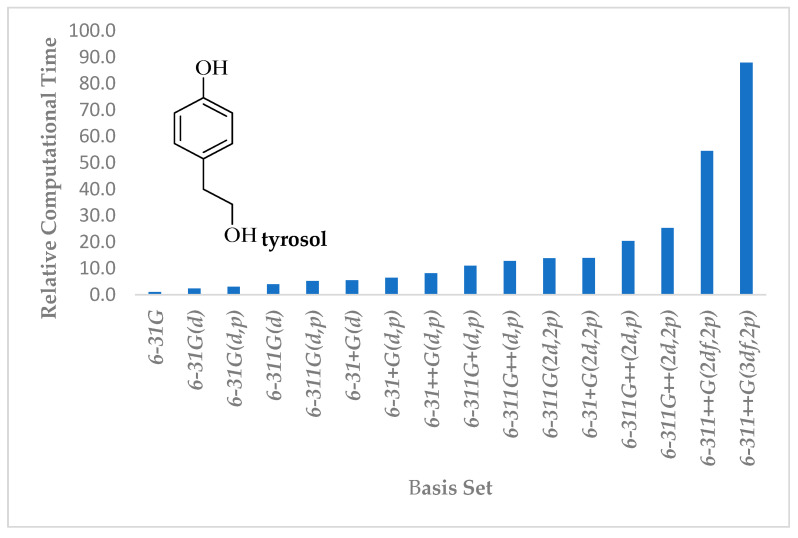
Relative computational time for structural optimization of tyrosol (including frequency calculation) in the gas phase using B3LYP and different basis sets used in radical scavenging studies (Nenadis, Pyrka, Tsimidou, this work).

The methodologies used in the literature can be classified into three groups: (a) semi-empirical methods (Austin Model 1, AM1, Parametrization Method 3 (PM3), 6 (PM6), etc.), (b) sophisticated methods (Hartree–Fock and density functional theory, DFT), and (c) their combination (e.g., DFT/AM1). The choice among the three methodologies is based on a multitude of factors, including the background knowledge of investigators, software package availability, and computer resources. A crucial element is the duration of computation. The latter is influenced by various factors such as the method selected, the size of the examined compound, the molecular species (parent compounds, anions, or neutral or ionized free radicals), the size of the basis set, that is, the group of functions used to describe the molecular orbitals, and whether the calculation will be carried out in the gas or the liquid phase. All these parameters affect the population of integrals that are needed to be solved and, therefore, have an impact on the required time/cost of the calculation (see as an example Figure 4). 

To decide whether a method is appropriate for calculations, some investigators examine the relative difference between the derived bond lengths and angle values of some of the compounds under study with experimental ones obtained with X-ray analysis (where available), which should not be higher than 2–3% [35]. Other researchers evaluate method performance through comparison between the derived and experimental value of an index, e.g., the gas-phase bond dissociation energy (BDEg) value, for a given compound, commonly for phenol, for which an experimental one is also available [36]. Lower values for these two molecular descriptors postulate a higher radical scavenging activity. The experimental BDE value for phenol is within the range of 85.1–90.2 kcal/mol [37,38], whereas, by consensus, the mean value of 88.7 kcal/mol can also be used [17]. Other typical approaches rely on the examination of the correlation between the computed values of an index with the experimental activity measurements for a set of compounds [39].

Density functional theory (DFT) calculations in combination—or not—with a semi-empirical structure optimization step dominate antioxidant activity studies [7,15,16,17,18,19,20,21,22]. Contrary to semi-empirical methods, which take into consideration only the valence electrons, DFT ones model all electrons in a molecule. The energy of the test compound can be determined from the electron density instead of a wave function. In this way, a substantial decrease in computation time in comparison to Hartree–Fock calculations is succeeded and with comparable accuracy to that of more demanding methods (e.g., Møller–Plesset, MP2) [40]. This is related to the improvements made to model the Coulombic interactions between the electrons of a system, achieved with the use of various approximations such as those included in hybrid functionals. Among the available functionals, the Becke 3 and Lee Yang Parr, B3LYP, is the most frequently employed one in DFT calculations of radical scavenging activity. According to Spiegel [7], the use of the respective functional accounted for ~47% of the studies, followed by M06-2X and M05-2X (∼26% and ∼17%, respectively). The author highlighted that these two functionals progressively replace the B3LYP based on their superior performance in various types of computations, including those involving free radicals, but despite some underestimations, the trend in activity using B3LYP is the same. Apart from the above, discrepancies can be found in the size of the basis set employed, which may be different even in the same research but for different purposes (e.g., structural investigation, thermochemistry). Searching the literature of the last decade, Spiegel [7] reported that a significant percentage (~36.5%) using the thermochemical approach prefer the 6-311++G(d,p) basis set, followed by 6-311 + G(d,p) (15.5%), with the other three in the range of 9.5 to 5.4%. Cost, as already reported previously, is a decisive factor, and large differences in the computational time may occur by moving from a typical split-valence double-zeta basis set (6-31G) to a triple and quadruple zeta, and further on by including polarization functionals (required to better describe highly polarized bonds) and diffuse ones to heavy and light atoms (being required to model, ionic species, and free radicals). This is better exemplified by the calculations carried out for tyrosol for the aim of the present study (Figure 4). Each column represents the relative time required for structure optimization in the gas phase (including frequency calculation) using various basis sets employed in the radical scavenging activity studies [7] toward that required using 6-31G. As evident, the cost increases more when polarization functionals are introduced compared to triple zeta, and computation becomes more costly by increasing the functionals for heavy atoms, rather than for the light ones. In a recent paper [41], the suitability of 6-311G(d,p) for overall antioxidant studies in terms of accuracy and cost has been reported, which on the basis of data shown in Figure 4, is not that time consuming; however, as stated by the authors, ‘its adequacy requires further substantiation’.

The methodology adopted almost two decades before was to carry out the DFT calculations in the gas phase, as a first step to screen the selected compounds and obtain an idea of the activity of a target compound as soon as possible [17]. Nevertheless, as reactions take place usually in the liquid phase, solute–solvent interactions have to be inevitably introduced, and this is carried out implicitly and/or explicitly. Implicit solvent models rely on the localization of the target compound in a cavity form within an electric field designed as a continuum having a dielectric constant value identical to that of the solvent. The dielectric field can polarize the molecule, which in turn polarizes the field (polarized continuum model). The result is explained by a charge distribution on the surface of the cavity. The solvation energy (ΔG_sol_) is usually the sum of the electrostatic component (ΔG_el_), the dispersion–repulsion interaction (ΔG_dr_), and the cavity formation (ΔG_cav_). A broad range of models is available that differ in the approximations [42,43]. The integral equation formalism variation of the polarizable continuum model (IEF-PCM) is a common one, with the solvation model based on density (SMD) being of increasing popularity and recommended as more suitable for kinetic studies [44]. The latter model, as well as others of the SMx family, considers both electrostatic and non-electrostatic contributions to Δ_solvG_, which is required for more accurate calculations of solvation energies [43]. 

These models usually provide a reliable description of solvation at the cost of a considerable rise in the computational period when contrasted to gas-phase calculations. Explicit solvent effects are valuable when the formation of intermolecular hydrogen bonds is favored, having, thus, an effect on hydrogen atom donation [45]. Following such an approach, individual molecules of solvent are incorporated in the input, forming hydrogen bonds at the appropriate sites of the molecule in the gas phase [46] or liquid phase to comprise also implicit solvent effects [47]. Such a selection, however, increases the cost of calculation markedly and, thus, is not the first choice of researchers [7].

At this point, it should be stressed that despite the lack of consensus on a common protocol for the calculation of the various indices, Galano and Alvarez-Idaboy [43] made a proposal, the so-called ‘Quantum Mechanics-based Test for ‘Overall Free Radical Scavenging Activity’ known as QM-ORSA. The proposed protocol is reliable for the study of radical–molecule reactions in the liquid phase. Among the various characteristics of the protocol, it should be noted that the quantitation of the antioxidant activity is either absolute (overall rate coefficient) or relative, using Trolox as a reference. The test also takes into account the influence of pH value and has been validated via comparison with experimental findings, proving that the error of prediction is no larger than the experimental one.

Last but not least is the modeling to predict the activity as a function of different parameters in the so-called structure–activity relationship (QSAR) analysis, which aims at identifying the most efficient antioxidants and assists the designing of new ones that could be more effective in reducing the cost and animal experiments. This methodology, described in the review by Alov et al. [18], often involves the development of multiparametric models, which may comprise several groups of descriptors such as electronic, hydrophobic, steric, constitutional, and topological. While the last three can be calculated from the 2D or 3D structure, the first two can also be obtained experimentally. Descriptors such as Log *P*, dipole moment, and polarizability are important ones, as lipophilicity determines to a great extent the presence of the compounds at the site of oxidation in a real system [48]. 

## 4. Phenolic Compounds of Olive Tree Products and By-Products

**Figure 5:**
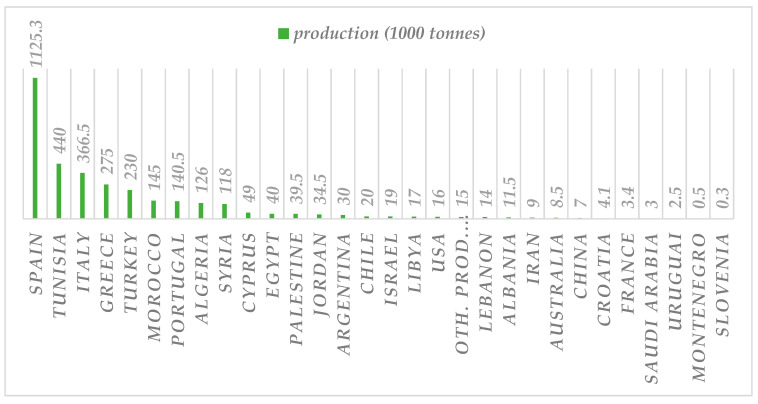
World olive oil production (×1000 tonnes) based on International Olive Council (IOC) statistics for 2019/20 [49,50].

The olive tree is the emblematic cultivation that thrives currently not only around the Mediterranean basin but in all continents and in both hemispheres, as it is illustrated in Figure 5 for olive oil production per country according to the International Olive Council statistics [49,50]. 

Raw olives are not consumed as such but are either processed by mechanical means to obtain the ‘virgin’ type of olive oil or are subjected to various treatments to yield the so-called table olives. Until very recently, the remaining solid mass after oil removal was used to produce lower-quality oil (pomace oil) with the aid of organic solvents. Seeds and the remaining pulp mass were used as fuel material. Recently, in line with the cyclic economy principles, the solid by-product has been viewed as an interesting source for the development of novel edible products [51]. Leaves and wastewater from the olive oil or the table olive industry are not recycled regularly or used as sources to recover bioactive compounds such as polar phenolic antioxidants, although research evidence is ample [52]. As demonstrated in the literature, the major lipophilic phenolic compound in olive tree products and by-products is α-tocopherol. Tsimidou [53], in a chapter dedicated to tocopherols from olive tree products from all over the world, pointed out that α-tocopherol is the major analog among the eight known forms of vitamin E (>90% of the total amount), whereas the remaining amount is comprised of β- and γ- forms. The most concentrated source in this phenolic type vitamer is high-quality extra virgin olive oil, irrespective of the olive cultivar. Its concentration in this rich in monounsaturated fatty acids (i.e., oleic acid) oil is important regarding recommendations for the daily intake of vitamin E [54], and it is affected by extraction and storage conditions [55]. Currently, most cultivars may yield virgin olive oils with more than 200 mg α-tocopherol/ kg oil. Table olives, preparations based on olives and olive oil, and olive leaves are important sources of α-tocopherol as well [5]. These materials have been reported to be among the richest sources from wild and crop plants of the Mediterranean region [56] due to their biosynthesis in chloroplast membranes within the plant’s green tissues. Theoretical calculations for the antioxidant activity of tocopherols are frequent in the literature [12,15,17,18,20] because these nutrients are of generic interest and are present in a variety of natural products, mainly of plant origin. On the opposite, the most characteristic phenolic antioxidants of olive tree products and by-products are the so-called ‘polar phenolic compounds’ because they are found in the polar fraction of olives, raw and processed, and olive oil and leaves, and are also transferred freely in pomace and wastewaters. The literature data [57,58,59,60,61,62,63,64,65,66,67,68,69,70,71,72,73,74,75,76] shown in Supplementary Materials Table S1 give an overview of the diversity of ‘polar phenolic compounds’ present in raw olives [57,62,67,69,71,76], virgin olive oil [60,61,62,65,69,71,72,73,74,75,76], table olives [66,67,68,75] and the major by-products (leaves, solid and liquid industrial streams) [58,59,62,63,64,66,69,70,71]. The catalogue of the different structures identified so far is rather long, i.e., 103 compounds to our knowledge. It is clear from the Supplementary Materials Table S1 that in the polar fraction of the olives, oil, and leaves, the secoiridoid type of compounds, their derivatives together with the related simple phenols hydroxytyrosol, tyrosol and their derivatives (Supplementary Materials Table S1, compounds No **1**–**30** and **90**–**101**, respectively), and isochromans (Supplementary Materials Table S1, compounds No **102**–**103**) prevail in number. Flavonoids (Supplementary Materials Table S1, compounds No **31**–**56**), lignans (Supplementary Materials Table S1, compounds No **57**–**60**), phenolic acids (Supplementary Materials Table S1, compounds No **61**–**83**, phenylethanoids (Supplementary Materials Table S1, compounds No **84**–**89**), and other phenolic compounds are also present, but their presence cannot be related exclusively with these natural sources. Given that oleuropein, ligstroside, hydroxytyrosol, tyrosol, and related compounds differentiate the olive tree products and by-products from other sources of primary antioxidants, the literature survey was focused on theoretical studies available for the prediction of their antioxidant activity. 

## 5. Computational Studies on the Radical Scavenging Activity of Olive Secoiridoids and Related Compounds

The antioxidant properties of the secoiridoids of olive tree products and by-products attracted the interest of the food industry for technological reasons [74,75] and of the medical community for a multitude of related effects, among which their contribution to the protection of blood lipids from oxidative stress has been authorized as a health claim for olive oil in the European legislation [77]. Interestingly, the computational studies available for them have been scarce since the first publication in 2005 [24,30,34,78,79,80,81,82,83,84,85,86,87,88,89]. This means 15 original articles over 17 years. 

Nenadis and collaborators [78] were the first to report BDE and IP values for 18 phenolic compounds, namely, tyrosol, tyrosol glycoside, ligstroside, the aldehydic form of ligstroside aglycone, the tyrosol-dialdehydic form of elenolic acid, hydroxytyrosol, hydroxytyrosol acetate, oleuropein, demethyloleuropein, four types of oleuropein aglycone, the hydroxytyrosol-dialdehydic form of elenolic acid, verbascoside, caffeic acid, and the lignans pinoresinol and 1-acetoxypinoresinol (Supplementary Materials Table S1, compounds No **4**, **6**, **10**, **11**, **13**, **17**, **18**, **22**, **23**, **56**, **60**, **70**, **84**, **94**–**96**, **98**). Considering the size of certain compounds (300–624 amu) and computing restrictions at the time of the study, the authors employed a semi-empirical method for structure optimization and frequency calculation in the gas phase, whereas to obtain the electronic energies, the B3LYP functional on the 6-31G (p’) level was used, modifying the normal p exponent of the polarization functions only on hydrogen to the value of 1.0 [90]. Focus was given to the determination of the hydrogen atom and electron donating properties considering HAT and SET mechanisms. The catechol derivatives, such as oleuropein and related compounds, presented lower BDE values (78.4 to 80.1 kcal/mol) compared to ligstroside and related monophenols (85.1 to 86.2 kcal/mol), and lower IP values in most cases (168.2–177.2 vs. 171.4–177.4 kcal/mol, respectively). The lack of conjugation in the side chain was predicted to result in less stable phenoxy radicals, as evident via the spin density calculation for oleuropein and hydroxytyrosol compared to that of caffeic acid. Even though no clear trend was observed in terms of IP values, and the observed marginal effect of the side chain characteristics on BDE values, the authors, using a fragmentation approach available in the ChemDraw program, highlighted the differences in lipophilicity (in terms of Log *P* values, 1.23 to −0.58 for ligstroside and derivatives, 1.64 to −1.24 for oleuropein and derivatives) indicating the water solubility (Log *P* values: −0.39 to −1.24) of the glucosides (tyrosol glucoside, ligstroside, oleuropein, dimethyl oleuropein, verbascoside) and the increase in lipophilicity upon hydrolysis (Log *P* values: 0.65 to 1.64). The latter property is of paramount importance for effectiveness in real systems containing lipids [91]. The authors also stressed the importance of planarity, which may facilitate penetration via lipid membranes. This feature did not characterize the tested compounds, especially the large ones, according to the geometry obtained. Although prediction findings were supported by the experimental evidence on the location of some of the compounds in liposome membranes according to Paiva-Martins et al. [92], agreement with experimental findings on activity trends was not always observed. The trend in activity for tyrosol, hydroxytyrosol, oleuropein, and caffeic acid in terms of BDE values according to [78] was in line with that for the % scavenging of DPPH^●^ obtained by the same group [93]. However, the significantly lower efficiency of oleuropein compared to caffeic acid and hydroxytyrosol during autoxidation of triolein (monitored through peroxides formation), carried out in parallel with the theoretical calculations [78] could not be supported in terms of BDE values or Log *P* ones. A discrepancy was also evidenced in terms of EC_50_ values for the scavenging of the same radical by Gordon et al. [94], who reported the order oleuropein aglycone (0.12) >> hydroxytyrosol (0.19) > oleuropein (0.22) > hydroxytyrosol acetate (0.25). Predictions in terms of BDE [77] were found to be closer to the experimental findings in autoxidized stripped olive oil (hydroxytyrosol ≥ hydroxytyrosol acetate ≥ oleuropein aglycone >>> oleuropein reported by Gordon et al. [94]. In the same theoretical work [78], the authors discussed some findings by Owen et al. [95] regarding the scavenging of hydroxyl radicals produced via a hypoxanthine/xanthine oxidase system. They proposed electron donation as the possible mechanism of scavenging by the studied compounds, including hydroxytyrosol, oleuropein, and caffeic acid, in terms of IP values, with the prerequisite of no interference of the molecules with the enzymes used. Regarding a set of hydroxytyrosol metabolites identified only in biological fluids (3,4-dihydrohyphenylacetaldehyde, 3,4-dihydrohyphenylacetic acid, homovanillic alcohol, aldehyde, and acid, monoglucuronide and monosulfate conjugates, 3-hyroxy-4-methoxyphenylacetic acid, and 3,4-dimethoxyphenylethanol) [96], it was evidenced that when the catechol moiety remained intact, then the radical scavenging efficiency was maintained, too. The latter decreased upon conversion to methoxylated forms and was diminished upon conversion to glucuronide or sulfate derivatives, and even more upon methoxylation of the *p*-hydroxyl group. The authors took into account published DPPH^●^ assay data [96] and suggested the re-evaluation of results as the almost 5-fold higher activity reported for the glucuronide compared to hydroxytyrosol was not supported by the computed IP values in the liquid phase. 

**Figure 6:**
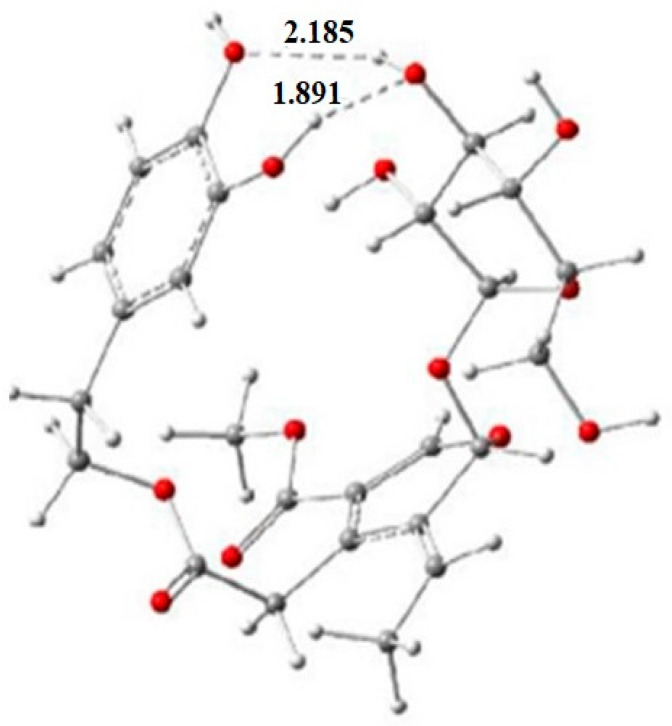
The intramolecular hydrogen bonds between catechol and sugar moieties and their bond lengths (Å) in the optimized geometry of 1d radical (the product of C3 H abstraction of oleuropein) adapted from [30].

Almost a decade later, theoretical calculations for the radical scavenging activity of oleuropein, ligstroside, oleuropein aglycone, oleuroside, 3,4-DHPEA-EA, oleacein, oleocanthal, hydroxytyrosol acetate, salidroside (see Supplementary Materials Table S1, no **4**, **9**, an isomer of **9**, **16**, **18**, **30**, **94**, **96**), plus chlorogenic acid were examined [29] to shed light on the effect of sugar and catechol moieties, and of the pyran and carbonyl groups to the antioxidant activity. For the purpose of that study, the B3LYP/6-31G (d,p) level of theory was used for optimization in the gas phase, but this time, calculations were also carried out in the liquid phase (benzene), adopting PCM. The authors provided optimized structures for the first time and highlighted that in the case of oleuropein, the conformation was expected to be such that sugar and catechol moieties could form intramolecular hydrogen bonds (Figure 6). The authors calculated various indices based on frontier orbital energy values and intrinsic molecular indices, namely BDE and IP ones. The BDE calculations showed that the active site was not located in the catechol moiety but at the C-3 in the elenolic acid moiety. The conformation found was proposed to stabilize the carbon radical formed so that the C-H BDE value was lower by ~3 kcal/mol than that of the aromatic O-H group at C-4. A similar prediction was made for the ligstroside.

For all the other compounds, the active site was found to be, as expected from experimental work data, in the aromatic ring. At this point, it should be highlighted that -C-H bonds in various compounds have been found as more abstractable than -O-H [97,98,99]. Such a finding may need further investigation as the barrier of reaction with free radicals has not been calculated, and theory suggests that this is rather large with C-H bonds, whereas it is negligible with phenolic O-H groups [99]. Hassanzadeh et al. [30] attributed the difficulty of oleuropein to react with the bulky DPPH^●^ to steric hindrance phenomena. They suggested that the reaction occurs at less active sights of the molecule, a fact that may explains the underestimated activity found. On the other hand, discussing the lack of scavenging activity of oleuropein toward some small-size ionized radicals (e.g., O_2_^●−^) experimentally, the same authors attributed such a finding to electrostatic repulsion due to the accumulation of similar charges close to the active site of the molecule. This was not observed for the non-charged NO^●^ and OH^●^. Such an explanation was in line with experimental findings published by Chimi et al. [100] and Czerwińska et al. [101]. 

The examination of the conformation of oleuropein has been the subject of other studies, which employed molecular dynamics in gas, or in liquid phase (water) and dispersed lipid system model (e.g., triolein–water system) to obtain more realistic results [79,80]. Refining of the most stable conformer was carried out using semi-empirical or advanced ab initio computations, and an appropriate software to design lipids, namely GROMACS [102]. The findings in the gas phase for the oleuropein structure, were in accordance with those reported by Hassanzadeh et al. [30]. However, in the case of modeling in the presence of water and triolein–water systems [80], extensive conformational changes were found. Specifically, in water, the molecule could adopt various conformations, namely a closed-like U form, and a semi-opened form, to an opened form characterized by high variations in the distance between glucose and hydroxytyrosol. In the triolein–water system, a more open form where the glucose moiety could be almost aligned with the hydroxytyrosol and elenolic acid moieties was observed. A similar publication is available for oleuropein aglycone [81], where the lipid membrane models, including phospholipids and cholesterol, were prepared using the Charmm-Gui web server [103]. In that work, the aglycone was found to be located between the hydrocarbon acyl chains of the phospholipids, but its specific location and molecular interactions were influenced by the lipid system. Thus, it was predicted to be closer to the membrane surface in the 1-palmitoyl-2-oleoyl-*sn*-glycero-3-phosphocholine/cholesterol system, but it was expected to be located deeper in the 1-palmitoyl-2-oleoyl-*sn*-glycero-3-phosphocholine/1-palmitoyl-2-oleoyl-*sn*-glycero-3-phospho-(1′-rac-glycerol)/cholesterol system. The aglycone was expected to interact stronger with 1-palmitoyl-2-oleoyl-*sn*-glycero-3-phosphocholine, suggesting specific interactions with negatively charged phospholipids. Aree and Jongrungruangchok [80] carried out a completely different study than those discussed so far. They examined the inclusion complexes of tyrosol, hydroxytyrosol, and oleuropein in β-cyclodextrin, which is a means to improve the radical scavenging activity of natural compounds. In fact, they characterized their study as a structure–radical scavenging activity one, and DFT, after starting with semi-empirical methods (PM3) for optimization, was used to calculate the thermodynamic stability of the complexes, which was found to be fairly in agreement with their DPPH radical scavenging efficiency. The ability of oleuropein to donate hydrogen atoms was examined again just recently by Baysal et al. [83] but without being the principal aim of the study. BDE and ionization energy values were computed using the B3LYP/6-31-g (d) level of theory for optimization and frequency calculation in the gas and liquid phase (methanol) to mimic the environment of the DPPH radical scavenging assay of olive leaf extracts. Calculations were carried out also for Trolox, which was used as a reference experimentally. According to the authors, this secoiridoid was considered better hydrogen and electron donor than Trolox, also showing that hydrogen atom donation was feasible from the C-H bond. This evidence agreed with the experimental evidence for the DPPH^●^ scavenging activity of the two compounds found by Gordon et al. [94], but not with that by Nenadis and Tsimidou [93] using the same assay.

Li et al. [84] studied tyrosol and hydroxytyrosol as well as some of their derivatives (dialdehydic form of decarboxymethyl elenolic acid linked to hydroxytyrosol or tyrosol, and hydroxytyrosol acetate; see Supplementary Materials Table S1 No **16**, **27**, **94**, **95**, **98**), as part of a wider group of antioxidants (coenzyme Q, flavonoids, curcumins, indolinonic hydroxylamines, phenothiazines, and edaravones, antioxidants used as food additives) having as a goal to show the accuracy of the ONIOM-G3B3 method for predicting the BDE values for different molecules. Employing the ONIOM (integrated molecular orbital approach), a molecule is divided first into different fragments. Then, various levels of theory, which differ in computational cost and accuracy, are applied to each of the fragments and to the whole molecule. This strategy aims at evaluating by extrapolation the results of the highest level of theory to the whole molecule. The highest level of theory used was the G3B3, which at the period of method development could work only for molecules with less than eight non-hydrogen atoms, as for larger ones, it was “too expensive and out of reach” [104]. As postulated, the computed values showed the superiority of the hydrogen atom donation of catechol derivatives, which was ~80 kcal/mol, ~8 kcal/mol lower than those calculated for tyrosol and the dialdehydic form of decarboxymethyl elenolic acid linked to tyrosol. Except for noting the hydrogen atom donation via the -OH at C-4 and the fact that olive phenols “are good antioxidants which take effect via hydrogen atom-transfer”, no discussion on experimental findings in the literature was made.

Hydroxytyrosol, the smallest in size molecule deriving from oleuropein, was first studied by Erkoc et al. [85], including its three radical isomers and three hydroxytyrosol-dihydroxyl isomers. Employing semi-empirical calculations (AM1), optimized structures were obtained, as well as values of frontier orbitals, heats of formation, and dipole moments. The authors commented on radical stability and polarity, which may affect the ability to scavenge peroxyl radicals in aqueous environments without proceeding with the calculation of other molecular indices. However, they mentioned that hydroxytyrosol and derived radicals were expected to be highly hydrophilic in terms of dipole moment values (1.74 to 3.66 D), suggesting that in this way, the aqueous peroxyl radical scavenging near the cell membrane may be feasible, as also shown experimentally in the past by Saija et al. [105]. 

One year later, the pair tyrosol (simple phenol deriving from ligstroside)/hydroxytyrosol was studied by Leopoldini et al. [86] using DFT. Simple phenol and a few other compounds were included in that study on the H-atom versus electron transfer mechanism. The optimization was carried out using B3LYP at a too-small basis set (6-31G) in the gas and liquid phases (benzene, water) to simulate real environments, but for single-point calculations, the too-large set 6-31++G(3df,2p) was also used. The authors showed that hydroxytyrosol was a more efficient hydrogen atom and electron donor than tyrosol, and compared to other compounds, the former was considered a very efficient hydrogen atom donor, but a mediocre electron donor. The differences were greater between the two compounds in the liquid phase but only regarding the IP values. An additional observation was that in the gas phase and benzene, its phenoxy radical from C-4 was more stable than that at C-3 (by 1.40 and 0.75 kcal/mol, respectively), whereas the opposite (difference of 0.76 kcal/mol) was found in water. Rezaei-Sadabady et al. [87], applying a semi-empirical method (AM1), showed the better activity of hydroxytyrosol and acetate over tyrosol in terms of gas-phase BDE values and designed two derivatives aiming at proposing novel antioxidants. Davalos et al. [88], using experimental methods to determine the onset temperature of melting point and enthalpy of fusion of tyrosol and hydroxytyrosol, using M05-2X at 6-311++G(d,p) level of theory to carry in parallel computation with composite ab initio methods (G3, G4), confirmed the exceptional agreement of theoretical with the experimental measurements. They exhibited that there is a stabilizing effect via H-bond formation between the hydroxyethyl chain and the aromatic ring (OH···π interaction), which is smaller in the formed radicals rather than in the parent molecules. Furthermore, they showed the accuracy of the computed BDE values and verified the superiority in antioxidant activity of hydroxytyrosol over tyrosol. Furthermore, mentioning different published works, and based on the BDE values, which were considered “an excellent primary indicator of antioxidant activity”, they further added that hydroxytyrosol should be more efficient than kaempferol, thymol, and homovanillic derivatives, of comparable efficiency to oleuropein, *p*-cresol, guaiacol, creosol, isoespintanol, and epicatechin, and less effective than gallic acid, resveratrol, and α-tocopherol.

Galano et al. [34] followed a different approach for the study of the pair tyrosol/hydroxytyrosol. Specifically, using the M05-2X functional and the 6-311+G(d,p) basis set for calculations in the liquid phase adopting the SMD continuum model and in pentylethanoate and water as media to mimic real conditions, they examined through the kinetic approach (rate constant calculation) the scavenging efficiency toward ^●^OCH_3_, ^●^OOH, ^●^OH, ^●^OCCl_3_, and ^●^OOCCl_3_ radicals in non-polar and polar media. They also examined the possibility of forming an adduct and the ability to act via HAT, SET, or SETP, including the branching ratios. Their findings pointed out that (i) the SET and SETP mechanism was not expected to contribute to the overall reactivity of the compounds to scavenge the two free radicals but could be important for the other three, and (ii) the hydrogen atom transfer was the predominant one so as a result hydroxytyrosol was found to be more efficient than tyrosol. Hydroxytyrosol was characterized as a good peroxyl scavenger, whereas tyrosol was a moderate one, with the reaction being faster in water than in non-polar media. In addition, they commented that HAT is expected to be dominant with every ^●^OR and ^●^OOR, with the prerequisite that R is an alkyl or an alkenyl group. In the same work, the authors computed the UV-vis spectra of the two compounds, their radical cations, and of reaction products for future comparisons with experimental techniques to confirm or decline their hypothesis on mechanisms of reaction.

Except for the above studies, Semidalas et al. [24] examined the antioxidant and anti-inflammatory activity of hydroxytyrosol exclusively. For the first activity, a series of calculations was performed using two ab initio (HF, MP2) and seven DFT (B3LYP, B3P86, B3PW91, PBE1PBE, M062X, wB97, and wB97X) methods. It was evidenced that better accuracy to structure matching of catechol with available crystallographic data was obtained at the wB97X 6-311G+(2d, 2p) level. The authors reported HAT to be the favorable mechanism of action in the gas phase, whereas SPLET was favored in water. The same year, Nenadis and Siskos [89] compared for the first time hydroxytyrosol with synthetic isochromans, one of which has been reported to form in olive oil upon extraction in small amounts and increase during oil aging [106]. The authors used 6-31G for conformational analysis and 6-311++G(2d,2p) for single-point energy calculation in the gas phase. For the specific compounds, data on their antioxidant activity using different experimental methods were already available in the literature [107]. The research data showed that hydrogen atom donation was preferable compared to electron donation. Moreover, it was found that a stepwise donation was feasible, leading, thus, to a quinone, followed by allylic hydrogen atom donation. Summing the total bond dissociation enthalpy values, because of multiple hydrogen atom donations, a high and statistically significant correlation was found with data obtained using the ABTS ^+.^ (*r* = 0.945, *p* = 0.004) and the ORAC assay (*r* = 0.991, *p* = 0.000).

**Figure 7:**
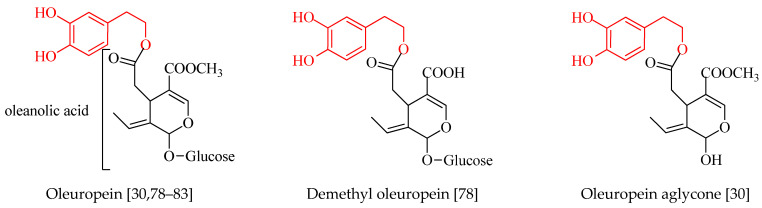

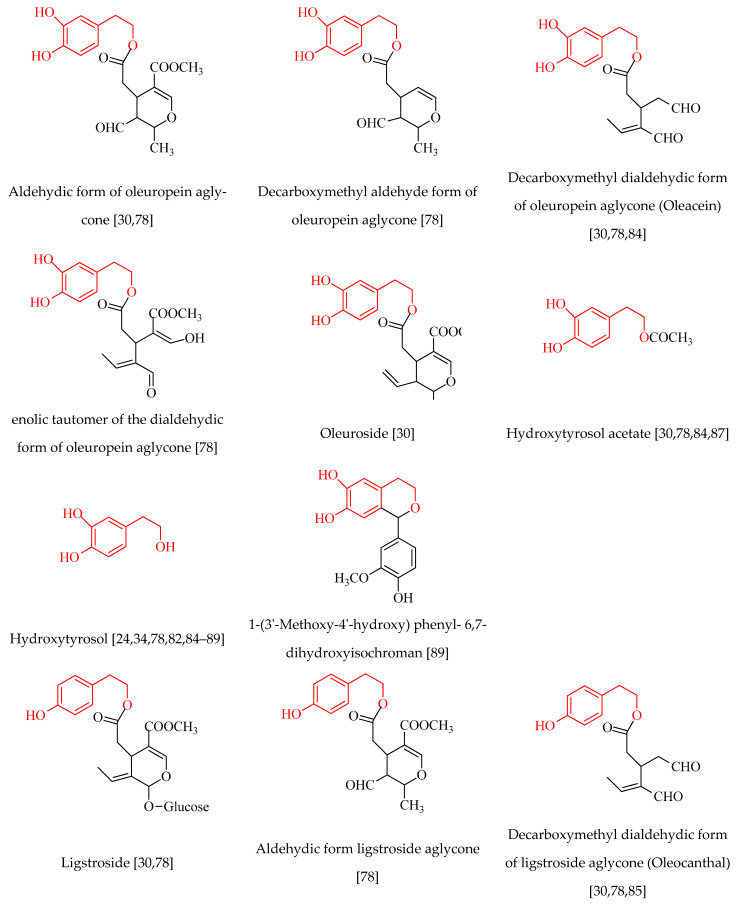

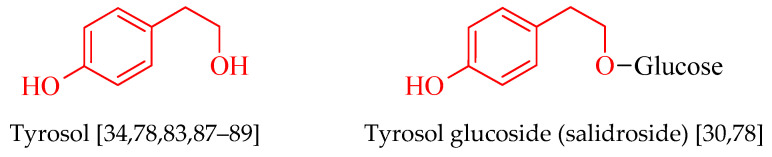
Oleuropein, ligstroside, and derivatives examined in literature for their radical scavenging activity using theoretical calculations.

The structural characteristics of the molecules examined using theoretical calculations so far, and the corresponding references, are depicted in Figure 7.

As is evident, the most investigated compound is hydroxytyrosol, followed by oleuropein, tyrosol, and hydroxytyrosol acetate. Oleuropein and ligstroside aglycones, such as oleacein and oleocanthal, which are expected to be found at high levels in fresh virgin olive oil, are less studied even though these compounds are also associated with biological effects. A single study took into consideration in vivo metabolites. Limited is also the array of descriptors or theoretical approaches that have been applied in most of the compounds so far, despite the technical improvements, availability of resources, and proposal of new and more complicated mechanisms (e.g., SPLHAT, double HAT, etc.). Thus, more work is needed considering the wide range of molecules identified so far in olive tree products and by-products, as shown in Supplementary Materials Table S1. This list is continuously updated as new structures are periodically discovered, although new acquisitions cannot be easily isolated due to their oxidative instability, limiting, thus, the feasibility of testing them experimentally. A characteristic example is the case of oleokoronal and oleomissional, or oleocanthalic and oleaceinic, acids (see Supplementary Materials Table S1, No **12**, **15**, **24**, **25**), which were recently identified in virgin olive oil [72,73].

## 6. Conclusions

In conclusion, our proposal is the continuation of the examination of the wide array of olive secoiridoids and related derivatives toward the construction of a database of antioxidant activity prioritization. The same should apply to in vivo identified metabolites, as structural changes may alter the redox properties of the parent compounds. This should be performed at least regarding the hydrogen atom and electron donation, with the most common theoretical indices of activity recognized in the benchmarking work of Wright et al. [12]. Toward a consensus for standardization of methodology, a step could be the inclusion of the same molecules as reference standards, e.g., phenol (generally considered as inactive or of poor activity) and/or Trolox (water-soluble analog of *ɑ*-tocopherol). Trolox is the most widely used reference compound in in vitro antioxidant activity studies and is recommended by international bodies for standardization of antioxidant activity methods. In this way, relative values (e.g., ΔBDE, ΔIP), an approach sporadically adopted by various researchers carrying out computational studies, may facilitate comparisons and communication of findings. Such a target will better be achieved if there is an agreement on an appropriate DFT functional, an average basis set (e.g., 6-311G(d,p)), considering the sizes of the identified compounds, and a solvation model (e.g., SMD), except for the gas phase, as this may influence the size of differences. Insertion of such a standardized protocol in any future theoretical antioxidant activity study, beyond the special needs of each research, will also make a feasible comparison with other natural classes of antioxidants. Mapping of antioxidants will lead to a solid characterization of strong hydrogen atoms and/or electron donors useful for researchers active in different disciplines.

## Figures and Tables

**Table 1:** Common molecular indices related to the radical scavenging potential of compounds and the formulae for their calculation.

Molecular Index	Formula *
Bond dissociation enthalpy (BDE)	H_r_ + H_h_ − H_p_
Adiabatic ionization potential (IP)	E_cr_ − E_p_
Proton dissociation enthalpy (PDE)	H_r_ + H_pr_ − H_cr_
Proton affinity (PA)	H_a_ + H_pr_ − H_p_
Electron transfer energy (ETE) **	E_r_ − E_a_

Where * H_r_: enthalpy of phenoxy radical, H_h_: enthalpy of the H-atom, H_p_: enthalpy of the parent molecule, E_cr_: energy of the cation radical, E_p_: energy of the parent molecule, H_pr_: enthalpy of the proton, H_cr_: enthalpy of the cation radical, H_a_: enthalpy of the anion, E_r_: energy of the phenoxy radical, E_a_: energy of the anion. (** In some publications, the enthalpies are considered, including also in the formula the enthalpy of the electron).

**Table 2:** Branching ratios (Γ) for the most important mechanisms and pathways involving hydroxytyrosol and methoxy radical in pentyl ethanoate and water (data abstracted from Table 2 of cited reference [34] and adjusted properly).

Structure and Numbering	Pentyl Ethanoate	Water
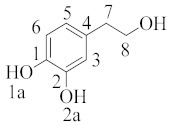 hydroxytyrosol	HAT(1a) = 61.53	HAT(1a) = 49.69
	HAT(2a) = 37.69	HAT(2a) = 49.69
	HAT(7) = 0.06	HAT(7) = 0.03
	HAT(8) = 0.14	HAT(8) = 0.10
	RAF*(1) = 0.55	RAF(1) = 0.20
	RAF(2) = 0.00	RAF(2) = 0.12
	RAF(3) = 0.00	RAF(3) = 0.01
	RAF(4) = 0.03	RAF(4) = 0.16
	RAF(5) = 0.00	RAF(5) = 0.01
	RAF(6) = 0.00	RAF(6) = 0.00

* RAF = radical adduct formation.

## Supplementary Materials

The following supporting information can be downloaded at https://www.mdpi.com/article/10.3390/molecules28052267/s1. Table S1: Secoiridoids and other phenolic compounds reported in the polar fraction of olive tree products and by-products.
