# Supplementary material for: The Contribution of Theoretical Prediction Studies to the Antioxidant Activity Assessment of the Bioactive Secoiridoids Encountered in Olive Tree Products and By-Products
Source: Molecules. 2023 Feb 28;28(5):2267. doi: 10.3390/molecules28052267 (PMC10005156; doi:10.3390/molecules28052267)
Supplement: Supplementary file 1 [file molecules-28-02267-s001.zip › molecules-2231686 SM.pdf]

Table S1. Secoiridoids and other phenolic compounds reported in olive tree products and by products*

No	Phenolic compounds	References	Molecular Mass	Olive Fruit	Olive Pulp/Mesocarp	Olive Seed/Stone	Virgin Olive Oil	Leaves	Olive Pomace	Olive Mill Wastewater, OMW
			**Secoiridoids**							
	**Oleuropein and related compounds**									
1	Oleuropein glucoside (di)	[57,65]	702.2					+		
2	Methoxy-oleuropein	[57,63]	570.5					+		
3	Hydroxy-oleuropein	[63]	556.2							
4	Oleuropein	[56,57,58,59,60,61,62,69, 70,73,75]	540.5	+	+	+		+	+	+
5	Oleuropein derivative	[63]	528.2					+		
6	Demethyl-oleuropein	[56,57,63,73,75,76]	526.5	+	+			+		
7	10-Hydroxy-oleuropein aglycone	[64]	396.4				+		+	
8	Oleuropein aglycone oxidized aldehyde and hydroxylic form/carboxylic form of oleuropein aglycone	[60,72]	394.1				+			
9	Oleuropein aglycone/3,4-DHPEA-EA	[58,59,61,63,70,73,74,75]	378.4	+			+	+	+	
10	Aldehydic form of oleuropein aglycone	[59,60,70]	377.4				+			
11	Dialdehydic form of oleuropein aglycone/Oleuropeindial	[60,64,73,74]	376.4				+			
12	Enolic tautomer of the dialdehydic form of oleuropein aglycone/Oleomissional	[64,71]	365.4				+			
13	Oleuropein aglycone derivative	[63]	360.1						+	
14	10-Hydroxy-decarboxymethyl oleuropein aglycone	[64]	336.1				+		+	+
15	Decarboxymethyl oleuropein aglycone oxidised dialdehyde form/Oleaceinic acid	[60,69,72]	336.1			+	+			
16	Dialdehydic form of decarboxymethyl elenolic acid linked to 3,4-DHPEA/Deacetoxy oleuropein aglycone/Oleacein	[59,60,61,63,73,74]	320.3				+			
17	Decarboxymethyl (deacetoxy) form of oleuropein aglycone	[64,70,73]	319.3				+			+
	**Ligstroside and related compounds**									
18	Ligstroside	[56,61,63]	524.5	+	+			+		
19	Ligstroside aglycone oxidized aldehyde and hydroxylic form/carboxylic form of ligstroside aglycone	[60,72]	378.1				+			
20	Methyl acetal of ligstroside aglycone	[66–68]	375.4	+						
21	Ligstroside aglycone/p-HPEA-EA	[57,59,63,70,73,74,75]	362.1				+	+		

22	Aldehydic form of ligstroside aglycone/Ligstral	[59,60,70]	361.4				+		+	+
23	Dialdehydic form of ligstroside aglycone/Ligstrodial	[60,64,73,74]	361.4				+		+	
24	Enolic tautomer of the dialdehydic form of ligstroside aglycone/Oleokoronal	[64,71]	361.4				+		+	+
25	Decarboxymethyl ligstroside aglycone oxidised dialdehyde form/Oleocanthalic acid	[60,72]	320.1				+			
26	Decarboxymethyl (deacetoxy) form of ligstroside aglycone	[64,73]	304.3				+			
27	Dialdehydic form of decarboxymethyl elenolic acid linked to p-HPEA/Oleocanthal/Deacetoxy ligstroside aglycone	[59,60,61,63,70,73,74]	304.3				+		+	
	**Other secoiridoids**									
28	Nuzhenide oleoside	[56]	1059			+			+	
29	Methyl (5E,6S)-5-ethylidene-4-[2-oxo-2-[[(2R,3S,4S,5R,6R)-3,4,5-trihydroxy-6-[2-(4-hydroxyphenyl)ethoxy]oxan-2-yl]methoxy]ethyl]-6-[(2S,3R,4S,5S,6R)-3,4,5-trihydroxy-6-(hydroxymethyl)oxan-2-yl]oxy-4H-pyran-3-carboxylate/Nuzhenide	[56,61,65]	686.2	+		+		+	+	+
30	Methyl 4-[2-[2-(3,4-dihydroxyphenyl)ethoxy]-2-oxoethyl]-3-ethenyl-2-[3,4,5-trihydroxy-6-(hydroxymethyl)oxan-2-yl]oxy-3,4-dihydro-2H-pyran-5-carboxylate/Oleuroside	[56,57,63]	540.5					+		
			**Flavonoids**							
31	Luteolin-7-O-rutinoside	[56,57,58,63,65]	610.5					+	+	+
32	Luteolin diglucoside	[57,59,65]	610.5					+	+	
33	(2S)-3′,5-Dihydroxy-4′-methoxy-7-[α-L-rhamnopyranosyl-(1→6)-β-D-glucopyranosyloxy]flavan-4-one/Hesperidin/Hesperetin 7-rutinoside	[56, 65]	610.6	+	+			+		
34	Quercetin rutinoside (3)/Rutin	[56,57,58,61,62,63,65,70,75]	610.5	+	+			+	+	+
35	Cyanidin-3-rutinoside	[66–68]	595.5							
36	Apigenin diglucoside	[57]	594.5					+		
	Apigenin rutinoside	[57,63,66–68,70]	578.5	+	+			+	+	
37	(2S,3R,4S,5S,6R)-2-[2-(3,4-Dihydroxyphenyl)-5,7-	[65,75]	484.8	+	+			+		

	dihydroxychromenylium-3-yl]oxy-6-(hydroxymethyl)oxane-3,4,5-triol/Cyanidin-3-glucoside									
38	Diosmetin-7-O-glucoside	[65]	462.4					+		
39	5-Hydroxy-2-(4-hydroxy-3-methoxyphenyl)-7-{[3,4,5-trihydroxy-6-(hydroxymethyl)oxan-2-yl]oxy}chromen-4-one/Chrysoeriol-7-O-glucoside	[57,63]	462.4					+		
40	Luteolin-4′-O-glucoside	[63]	448.4					+	+	
41	Luteolin-5-O-glucoside	[61,70]	448.4	+						
42	Luteolin-7-O-glucoside	[56,58,60,61,63,65,70,75]	448.4	+	+			+	+	+
43	Quercetin-3-rhamnoside	[66─68]	448.4	+						
44	Apigenin-7-O-glucoside	[56,57,61,63,65,70]	432.4	+	+			+	+	
45	3,4′,5,7-Tetrahydroxy-3′-methoxyflavone/Isorhamnetin	[57]	316.3							
46	4′,5-Dihydroxy-6,7-dimethoxyflavone/Cirsimaritin	[65]	314.3					+		
47	Gallocatechin	[57]	306.3					+		
48	(2R,3R)-3,3′,4′,5,7-Pentahydroxyflavan-4-one/Taxifolin	[59,63,70]	304.3				+	+		
49	3,3′,4′,5,7-Pentahydroxyflavone/Quercetin	[56,57,58,63]	302.2		+			+		+
50	5,7,3′-Trihydroxy-4′-methoxyflavone/Diosmetin	[57]	300.3					+		
51	(2R,3S)-2-(3,4-Dihydroxyphenyl)-3,4-dihydro-2H-chromene-3,5,7-triol/Catechin	[57,65]	290.3					+		
52	2-(3,4-Dihydroxyphenyl)-5,7-dihydroxychroman-4-one/Eriodictyol	[63]	288.3					+		
53	3′,4′,5,7-tetrahydroxyflavone/Luteolin	[56,57,58,59,61,62,63,65]	286.2		+		+	+	+	+
54	(2S)-4′,5,7-Trihydroxyflavan-4-one/Naringenin	[62]	272.3						+	
55	4′,5,7-trihydroxyflavone/Apigenin	[56,57,58,59,61,63]	270.3				+	+	+	+
56	Luteolin-hexoside	[63]	448.1					+	+	
			**Lignans**							
57	4,4′-[(1R,3aS,4R,6aS)-tetrahydro-1H,3H-furo[3,4-c]furan-1,4-diyl]bis(2,6-dimethoxyphenol)/Syringaresinol	[65]	418.4					+		
58	[(3R,3aS,6S,6aR)-3,6-bis(4-hydroxy-3-methoxyphenyl)-3,4,6,6a-tetrahydro-1H-furo[3,4-c]furan-3a-yl] acetate/1-Acetoxypinoresinol	[58, 60, 62, 69, 74]	416.4		+	+	+		+	
59	1-Hydroxypinoresinol	[59,70]	372.4				+			

60	4-[(3S,3aR,6S,6aR)-6-(4-Hydroxy-3-methoxyphenyl)-1,3,3a,4,6,6a-hexahydrofuro[3,4-c]furan-3-yl]-2-methoxyphenol/Pinoresinol	[58,59,61,63,70,75]	358.4		+	+	+	+	+	+
	**Phenolic acids**									
61	*p*-Coumaroyl-6'-secologanoside/Comselogoside	[62]	536.2							
62	(1S,3R,4R,5R)-3-{[(2E)-3-(3,4-dihydroxyphenyl)prop-2-enoyl]oxy}-1,4,5-trihydroxycyclohexane-1-carboxylic acid/Chlorogenic acid/Caffeoyl-quinic acid	[56,57,60,61,62,63,65,70,75]	354.3	+	+			+	+	
63	(2R,3R)-2-{[(2E)-3-(3,4-Dihydroxyphenyl)prop-2-enoyl]oxy}-3-hydroxybutanedioic acid/Caftaric acid	[65]	312.2					+		
64	(2E)-3-(4-Hydroxy-3,5-dimethoxyphenyl)prop-2-enoic acid/Sinapinic acid	[56,58,59,61,73,75]	224.2	+			+			+
65	4-Hydroxy-3,5-dimethoxybenzoic acid/Syringic acid	[56,57,58,61,65,70,73,74,75]	198.2	+	+		+	+		+
66	3-(4-Hydroxy-3-methoxyphenyl)-2-propenoic acid/Ferulic acid	[56,57,59,60,61,62,63,65,70,73,75]	194.2	+	+		+	+	+	
67	3,4-Dimethoxybenzoic acid/Veratric acid	[56,58,63]	182.2		+					+
68	(4-hydroxy-3-methoxyphenyl)acetic acid/Homovanillic acid	[56,59,62,65,73,75]	182.2	+	+		+	+	+	
69	3-(3,4-Dihydroxyphenyl)propanoic acid/Dihydrocaffeic acid	[59,70,75]	182.2	+	+		+			
70	3-(3,4-Dihydroxyphenyl)-2-propenoic acid/Caffeic acid	[56,57,58,59,60,61,62,63,65,73,74,75]	180.2	+	+		+	+	+	+
71	3,4,5-Trihydroxybenzoic acid/Gallic acid	[56,57,58,59,60,61,63,64,70,73,74]	170.1	+	+		+	+	+	+
72	4-Hydroxy-3-methoxybenzoic acid/Vanillic acid	[56,57,59,60,61,62,63,70,73,74,75]	168.2	+	+		+	+	+	+
73	3,4-Dihydroxyphenylacetic acid/Homoprotocatechuic acid	[59,70,75]	168.2	+	+		+			
74	(2E)-3-(3-Hydroxyphenyl)prop-2-enoic acid/m-Coumaric acid	[80]	164.2	+	+					
75	(2E)-3-(2-Hydroxyphenyl)prop-2-enoic acid/o-Coumaric acid	[55, 58, 59, 60, 72, 73, 74]	164.2	+	+		+			
76	(2E)-3-(4-Hydroxyphenyl)prop-2-enoic acid/p-Coumaric acid	[56,57,58,59,60,61,62,63,73,74,75]	164.2	+	+		+	+	+	+
77	2,5-Dihydroxy benzoic acid/Gentisic acid	[59,73]	154.1				+			
78	2,4-Dihydroxy benzoic acid/β-Resorcylic acid	[70]	154.1							

79	2,6-Dihydroxy benzoic acid/γ-Resorcylic acid	[70]	154.1							
80	3,4-Dihydroxybenzoic acid/Protocatechuic acid	[56,57,58,59,60,61,62,63,73,74,75]	154.1	+			+	+	+	+
81	4-Hydroxyphenylacetic acid	[56,57,59,70,73,75]	152.2	+	+		+			
82	3-Hydroxybenzoic acid/m-Salicylic acid	[59]	138.1				+			
83	4-Hydroxybenzoic acid/p-Salicylic acid	[56,57,58,60,61,62,63,73,74,75]	138.1	+			+	+	+	+
	**Phenylethanoids**									
84	[(2R,3R,4R,5R,6R)-6-[2-(3,4-Dihydroxyphenyl)-2-hydroxyethoxy]-5-hydroxy-2-(hydroxymethyl)-4-[(2S,3R,4R,5R,6S)-3,4,5-trihydroxy-6-methyloxan-2-yl]oxyoxan-3-yl] (E)-3-(3,4-dihydroxyphenyl)prop-2-enoate/β-hydroxy verbascoside/β-Hydroxyacteoside/Campneoside II	[58]	640.6							+
85	Diastereosiomer-β-hydroxy verbascoside	[58]	640.6							+
86	2-(3,4-Dihydroxyphenyl)ethyl *α*-L-rhamnopyranosyl-(1→4)-{5-O-[(2E)-3-(3,4-dihydroxymethyl)prop-2-enoyl]-β-D-glucopyranoside}/Verbascoside/Acetoside	[56,57,58,61,63,65,69,70,73]	624.6	+	+	+		+	+	+
87	Isoverbascoside	[57,58]	624.6					+		+
88	Oxidized isoverbascoside	[58]	624.2							+
89	Oxidized verbascoside	[58]	624.2							+
	**Simple phenols**									
	**Hydroxytyrosol & derivatives**									
90	Hydroxytyrosol diglucoside	[63]	478.2						+	
91	Hydroxytyrosol glucoside (4-O, 1-O, 3'-O, 7)	[57,58,61,62,70,74,75]	316.3	+			+	+		+
92	Hydroxytyrosol hexoside	[65]	316.1					+		
93	Methyl malate-B-hydroxytyrosol ester	[66-68]	284.1	+						
94	4-(Acetoxyethyl)-1,2-dihydroxybenzene/Hydroxytyrosol acetate	[63,65,70,74,75]	196.2					+	+	
95	Hydroxytyrosol/3,4-DHPEA/(3,4-dihydroxyphenyl) ethanol	[56,57,58,59,60,61,62,63,73,74,75]	154.2	+	+	+	+	+	+	+
	**Tyrosol & derivatives**									
96	Tyrosol glucoside/Salidroside	[56,57,62,63,65]	300.3		+	+		+	+	+
97	Tyrosol acetate/(*p*-hydroxyphenyl)ethyl acetate	[60,70,74]	180.2				+			
98	Tyrosol/*p*-HPEA-EA/(*p*-hydroxyphenyl)ethanol	[56,57,58,59,60,61,62,63,73,74,75]	138.2	+	+	+	+	+	+	+
	**Other**									

99	3,4-Dihydroxyphenylglycol/4-(1,2-dihydroxyethyl)benzene-1,2-diol	[58,62,63,70,75]	170.2	+			+	+
100	4-Hydroxy-3-methoxybenzaldehyde/Vanillin	[57,65,74]	152.2		+	+	+	+
101	Benzene-1,2-diol/Catechol	[58]	110.1					+
			**Hydroxy-isochromans**					
102	1-(3′-Methoxy-4′-hydroxy)phenyl-6,7-dihydroxyisochroman	[59,7]	288.3		+			
103	1-Phenyl-6,7-dihydroxyisochroman	[59,70]	242.3		+			

*Within each category compounds 1-103 are presented on descending molecular mass order
